# Editorial: Omics and its integration: a systems biology approach to understanding plant physiology

**DOI:** 10.3389/fpls.2023.1324901

**Published:** 2023-11-07

**Authors:** Kashif Ali, Milen I. Georgiev

**Affiliations:** ^1^ Department of Biosciences, Faculty of Life Sciences, Shaheed Zulfikar Ali Bhutto Institute of Science and Technology, Karachi, Pakistan; ^2^ Institute of Microbiology, Bulgarian Academy of Sciences, Plovdiv, Bulgaria

**Keywords:** omics, genomics, transcriptomics, proteomics, metabolomics, systems biology

The comprehension of intricate and interconnected biological processes necessitates the adoption of an integrative systems biology approach. The incorporation of diverse omics data, encompassing genomes, proteomics, transcriptomics, and metabolomics, will yield a comprehensive perspective that elucidates the interplay between different biomolecules and their roles in plant development, resilience, characteristics, and other significant attributes. The integration of multi-omics data has been made possible by recent advancements in analytical capabilities and the processing of massive data sets. As a result, researchers now possess a significantly enhanced understanding of the cell, tissue, organ, or even the entirety of an organism under investigation.

The primary objective of this Research Topic is not solely limited to the dissemination of the omics data that is of superior quality and dependability, but also encompasses the broader inquiry into the methods of integrating and establishing correlations amongst diverse data sets derived from numerous omics investigations. The studies included in this topic are focused on the areas of integration of multiple omics data for discovering gene(s) functions in autophagy, circadian clock and its connection with response against herbicides, resistance in plants, advancements in single cell and spatial transcriptomics, study of cultivars based on chemotype, and the interaction of proteins with the metabolites.

The emergence of single-cell and spatial transcriptomics has shifted the focus of researchers from the study of multicellular systems to the exploration of single-cell phenomena and spatial information. The utilization of single-cell transcriptomes enables the examination of transcriptomic profiles at the individual cell level, facilitating a deeper understanding of cellular heterogeneity. Conversely, spatial transcriptomes have the advantage of preserving and analyzing the spatial organization of gene expression patterns within tissues or organisms. Despite the usefulness and maturity of these two omics methods, additional study is required to assure their broad usage in plant studies. In a comprehensive review, Chen et al. conducted a comparative analysis of experimental methodologies employed in several plant species, focusing specifically on plant single-cell or spatial transcriptomics. The constraints and difficulties associated with single-cell and spatial transcriptome investigations are readily apparent, including restrictions in terms of application, geographical information, and resolution. The review also proposes additional applications, including the examination of roots at the single-cell level through cross-species analysis and asserts that the integration of single-cell transcriptome analysis with other omics studies is necessary to surpass the capabilities of individual omics analyses.

Discovering gene functions has been argued to be possible through the use of a systems biology technique, which combines molecular data from many levels of genome expression (*i.e.*, multi-omics data). Ding et al. analyzed the effects of mutations in two AuTophaGy-related (ATG) genes of Arabidopsis by combining lipidomics, metabolite mass-spectral imaging, and transcriptomics data from leaves and roots. The autophagy process, which is necessary for the degradation and recycling of macromolecules and organelles, is inhibited in the atg7 and atg9 mutants studied here. A prior knowledge of the exact biochemical function of the relevant proteins greatly facilitates the development of a comprehensive physiological model.

Plants have developed circadian clock mechanisms that facilitate the synchronization of biological processes with cyclic variations in the surrounding environment. Nevertheless, the interaction between the circadian clock and the herbicide response in rice remains unexplored. An interesting study by Chen et al. employed multi-omics data to elucidate the interplay between the circadian clock and herbicide response mechanisms in rice, specifically focusing on the epigenomic, transcriptomic, and metabolomic levels. Ultimately, it has been determined that the use of herbicides has the potential to impact the expression of several key oscillator genes inside the circadian clock of rice. This study can provide a theoretical framework for the development of novel herbicides or the cultivation of herbicide-resistant crops.

An interesting study by Zhang et al. capitalizes on the power of genomics and metabolomics for screening endophytic fungi with antibacterial potential, in *Rosa roxburghii* Tratt. This study identified 54 endophytes from *R. roxburghii* using molecular identification. Genomic, non-target metabolomic, and comparative genomic investigations examined the screened-out endophytic fungus’ biosynthesis.

Commercial distillation of mint produces essential oils, which are used in many consumer products. Most study on terpenoid oil constituent development has focused on leaves. Lange et al. found that mint species, including peppermint (*Mentha ^x^ piperita* L.), spearmint (*Mentha spicata* L.), and horsemint (*Mentha longifolia* (L.) Huds.), emit volatiles from stems, rhizomes, and roots. The terpenoid volatile composition of these organs differs significantly from leaves, suggesting significant chemical diversity exists. The newly sequenced *M. longifolia* CMEN 585 genome was used to identify candidate genes for monoterpene synthases (MTSs), which catalyze the first step in the biosynthesis of monoterpenoid volatiles. This was done to investigate the genetic and biochemical basis of chemical diversity. These MTSs can contribute to the production of all main monoterpene skeletons seen in volatiles from various mint organs.

Wheat is well recognized as a prominent staple crop within the global food production system. Nevertheless, the presence of stripe rust fungus has a substantial negative impact on both the productivity and quality of wheat. The findings of a study by Liu et al. provide valuable insights into the regulatory networks involved in the interactions between wheat and *Puccinia striiformis* f. sp. *tritici* (Pst). These insights have the potential to facilitate the development of durable resistance breeding strategies in wheat, which could contribute to addressing global environmental and food challenges. The findings of the study indicated that the infection caused by Pst led to the upregulation of genes and metabolites associated with the manufacture of phenylpropanoids. The unique resistance exhibited by R88 is controlled by the selective expression of genes that are involved in the precise regulation of interactions between wheat and Pst. In addition, the examination of the metabolome indicated that the accumulation of metabolites associated to lignin production was significantly influenced by Pst.

A major obstacle to widespread use of plant fibers is understanding the molecular components that affect the mechanical properties of both elementary and scutched fibers. Chabi et al. studied genome-wide transcription profiling in bast fiber-bearing tissues of seven flax cultivars and found 1041 differentially expressed genes, with 97 connected to cell wall metabolism. Three distinct clusters were identified based on gene expression levels for 32 proteins, out of which 4 proteins significantly linked with morphometric characteristics. These findings identify molecular players involved in determining in-planta fiber morphometrics and ex-planta fiber mechanical characteristics, which are crucial for determining basic and scutched fiber quality in flax.

Fernandes et al. assessed the phenotypic and chemotype of three high-THCA and one high-CBDA cultivars in their diploid, triploid, and tetraploid states using agronomic and metabolomic methods. This study reports that plant morphology showed a cultivar-dependent increase in height and leaf size with higher ploidy levels. Higher ploidy levels severely impacted cannabinoids, with concentrations of total cannabinoids, THCA, CBDA, and CBGA dropping significantly across all four cultivars. Overall, plant morphology observations match the giga phenotype in other polyploid plant species. This study indicated that tetraploidization can boost *Cannabis sativa* L. therapeutic potential, although cultivar and genotype-dependently. This effort prepares the breeding, biotechnological, and pharmaceutical industries to improve, evaluate, and use *C. sativa* chemical diversity.

Chlorophylls and carotenoids, which are generated from isoprenoid precursors, are needed for photosynthesis and plant greening. A study by Wolters et al. used *in silico* analysis, gene overexpression, transcriptomics, and metabolic profiling to study the impact of a homologous protein in the Russian dandelion (*Taraxacum koksaghyz*) on its rich isoprenoid network. Research suggests that TkPEL-like negatively controls genes involved to photosynthesis and chlorophyll in a light-dependent way, which is conserved across animals. These findings will guide future research on leaf isoprenoid biosynthesis and photomorphogenesis regulation, enabling breeding techniques to improve plant isoprenoid profiles and create plant-based industrial platforms.

The Guest Editors would like to express their gratitude to all the authors and reviewers of this Research Topic, and acknowledge their hard work and dedication toward the area of omics and systems biology. The Guest Editors believe that the presented researches will encourage the generation of more knowledge and valuable research in the fields of omics, their integration, and systems biology.

## Author contributions

KA: Writing – original draft. MG: Writing – review & editing.

